# Facile Control of Structured ZnO Polymeric Nanoparticles through Miniemulsion Polymerization: Kinetic and UV Shielding Effects

**DOI:** 10.3390/polym13152526

**Published:** 2021-07-30

**Authors:** Narissara Sudjaipraparat, Teeraporn Suteewong, Pramuan Tangboriboonrat

**Affiliations:** 1 Department of Chemistry, Faculty of Science, Mahidol University, Rama 6 Road, Phyathai, Bangkok 10400, Thailand; Narissara.sudjaipraparat@gmail.com; 2 Department of Chemical Engineering, School of Engineering, King Mongkut’s Institute of Technology Ladkrabang, Ladkrabang, Bangkok 10520, Thailand

**Keywords:** ZnO polymeric particle, core-shell, Pickering-like morphology, hollow particle, UV shielding, coating

## Abstract

Zinc oxide polymeric nanoparticles (ZPPs) of poly (styrene-co-acrylic acid) P(St/AA), containing oleic acid modified zinc oxide nanoparticles (OA-ZnO NPs), were synthesized via miniemulsion polymerization. By simply adjusting the quantity of reactants, i.e., sodium dodecyl sulfate (SDS) surfactant, potassium persulfate (KPS) initiator, and divinyl benzene (DVB) crosslinking agent, the location of ZnO NPs were altered from the inner (core) to the outer (shell), leading to core-shell and Pickering-like morphologies, respectively. The Pickering-like ZPPs were obtained when using SDS at below or equal to the critical micelle concentration (CMC). At above the CMC, the complete encapsulation of OA-ZnO NPs within the ZPPs depicted a kinetically controlled morphology. The transition to Pickering-like ZPPs also occurred when reducing the KPS from 2 to 0.5–1%. Whereas the DVB accelerated the polymerization rate and viscosity in the growing monomer-swollen nanodroplets and, hence, contributed to kinetic parameters on particle morphology, i.e., an increase in the DVB content increased the rate of polymerization. A hollow structure was obtained by replacing styrene with the more hydrophilic monomer, i.e., methyl methacrylate. All ZPPs-incorporated poly (vinyl alcohol) (PVA) films greatly improved shielding performance over the UV region and were relatively transparent on a white paper background. Due to the large number of ZnO NPs in the central region and, hence, the ease of electron transfer, composite films containing core-shell ZPPs possessed the highest UV blocking ability. ZnO NPs in the outer part of the hollow and Pickering-like ZPPs, on the other hand, facilitated the multiple light scattering according to the difference of refractive indices between the inorganic shell and organic/air core. These results confirm the advantage of structured ZPPs and their potential use as transparent UV shielding fillers.

## 1. Introduction

Zinc oxide nanoparticle (ZnO NP) is one of the most promising materials and has been widely used in various applications, such as chemical sensors, photovoltaic solar cells, piezoelectric/luminescent devices, textiles, and catalysts [1]. Apart from its wide band gap (3.4 eV) and large exciton binding energy (60 meV), other attractive properties of this low-cost semiconductor include biosafety, antibacterial activity, UV shielding, white appearance, and anti-corrosion. Similar to the majority of traditional inorganic UV absorbers, ZnO NPs possess high surface energy and often agglomerates, which deteriorates the aforementioned performances. However, the development of transparent UV-shielding materials, concerning solid NPs with high dispersion, is required [2,3]. Properties of these NP-incorporating shielding materials are mainly determined by the types of NP, the particle size, and the morphology. Core-shell structured NPs, especially the type of core/shell, the shell thickness, as well as the difference in the refractive indices (Δn) between the interior and the solid shell, all affect the shielding efficiency [4,5]. Absorption or transmission measurement can assess the shielding performance of NPs when applied in coating. The advantages of hollow NPs, an air void surrounded by a solid shell possessing lower density, multi-light scattering, and a high surface area, are therefore mentioned [5]. Generally, to lower cohesive forces between ZnO NPs, their surface is modified, mainly via two processes; (i) chemical adsorption of small capping and coupling agents, e.g., 3-(trimethoxysilyl)-propyl methacrylate (MPS), 3-aminopropyltriethoxysilane (APTES), and oleic acid (OA), and (ii) grafting with polymers, e.g., poly (methacrylic acid) [1,6,7]. These surface modifications further allow for the preparation of ZnO polymeric nanoparticles (ZPP), i.e., a polymer particle containing ZnO NPs [8,9,10]. As with other inorganic NPs, e.g., magnetic (M) NPs, not only does the content of the NP influence the property of the hybrid material, but so too does their position in the composite polymer particle. The polymer shell of magnetic microsphere is of vital importance for the composite to function in the biological conditions that require low toxicity, biocompatibility, and specific bioactive functionalities. ZnO-polystyrene (PS) core-shell particles, with high stability and good dispersion, provided perfect antibacterial film, which would be potentially useful in the field of coatings [9], whereas Janus and yolk-shell hollow PS-ZnO particles, having quantum dots as the co-seeds with PS, showed stable and strong fluorescence [10]. Due to the high sensitivity of ZnO NPs, as a Pickering stabilizer of the PS core, these ZPPs could react with either acid or base and, hence, could be a good pH adjusting material [11]. Among various processes for fabrication of ZPPs, the miniemulsion polymerization (mini-EP) is one of the most effective, facile, and straightforward ways to produce spherical particles with a readily controllable morphology. The mini-EP process provides the ability to control the particle size via the formation of miniemulsion (regarded as individually acting nanoreactors) and to nucleate all the droplets, containing NPs, of similar size and composition [12].

To achieve the desired architecture, relevant parameters, e.g., type and amount of surface modifiers of ZnO NPs that influenced the size and morphologies of the ZPPs, were focused on. By using ZnO NPs modified with various amounts of OA (OA-ZnO NPs), different morphologies of ZPPs made of PS were obtained. With a larger OA content (47 wt%; determined from thermogravimetric analysis (TGA)), yolk-shell ZPPs was formed because of the more uniform dispersion of OA-ZnO NPs in the St emulsion [10]. Whereas a Janus-like structure was obtained when using OA-ZnO NPs with a lower OA content (11 wt%). ZnO NPs coated with 0.1 wt% MPS (wt/wt MPS/ZnO colloid) were not miscible in styrene (St) emulsion and, hence, the PS core coated with ZnO NPs as Pickering-like particles were produced. The higher MPS content of 0.6–2 wt%, improving the compatibility between MPS-ZnO NPs and St, caused the encapsulation of ZnO in PS particles [13]. Using potassium persulfate (KPS) as an initiator, 70–96% of ZnO was entrapped in poly (styrene-*co*-methyl methacrylate) (P(St/MMA)) particles by the co-encapsulation of MPS-ZnO NPs and oil soluble organic sunscreen [14]. Up to 95% of the ZnO in the PS particles was attained when using APTES as the surface coupling agent and hexadecane (HD) as the hydrophobe [9]. The ZnO-embedded P(St/MMA) particles, using an azobisisobutyronitrile (AIBN) initiator and stearic-modified ZnO for the fabrication of multi-phase core materials, were also reported [15]. The size ratio of the ZnO NPs to the MMA/butyl acrylate monomers droplet affected their distribution and provided a composite copolymer with different morphologies [16]. The role of these parameters on the resulting morphologies often related to the chemical compatibility and site of polymerization [11,12,16]. What should be emphasized, such as kinetic and thermodynamic contributions, are not stated. Moreover, the role of crosslinking agent, e.g., divinyl benzene (DVB), on the ZPPs structure is less mentioned despite of the fact that DVB contributed to the rate of polymerization and affected the morphology of magnetic polymeric (MP) NPs [17]. MNPs were completely incorporated into PS or PMMA/DVB/acrylic acid (AA) due to the high viscosity caused from the presence of DVB inside the particle, which retained the MNPs from moving outward. By using the soap-free seeded EP with the Fe_3_O_4_ colloidal nanocrystal clusters as the seed, the morphology of the composite PS microspheres could be controlled from raspberry- and flower-like shapes to eccentric structures by adjusting the feeding weight ratio of the seed to the St monomer and varying the amount of DVB. Crosslinked MPS-Fe_3_O_4_@PS microspheres showed much more intact and smooth polymer layers with an increased DVB feeding amount. Recently, in the preparation of hollow magnetic polymeric particles (HoMPs) via the one-pot mini-EP, the location of OA-MNPs at the inner shell and void interface and in the shell layer was designed by adjusting the ratio of MMA/DVB, which caused the difference in polymerization kinetics and phase separation [18]. Since the polymerization took place when oligoradicals, generated in the continuous phase, entered the droplets consisting of OA-MNPs, MMA/DVB, and HD, varying the ratio of MMA/DVB, it altered the polarity of polymer the shell, which affected the partitioning of OA-MNPs at different locations in the final particle, i.e., the MNPs-rich at the inner wall and at the shell.

Herein, we reported a facile and direct method that can kinetically control the morphologies of ZPPs from Pickering-like to occluded-typed (core-shell) via the use of mini-EP of P(St/AA) using a KPS initiator. ZnO NPs, prepared via the co-precipitation method, were stabilized with OA and the effects of synthesis parameters, i.e., the amount of SDS surfactant, KPS initiator, and DVB crosslinking agent, on size and morphologies of ZPPs were elucidated using transmission electron microscopy and field emission scanning electron microscopy. The effect of a more hydrophilic monomer, i.e., MMA, in the presence of various amounts of DVB, on the morphological transition of ZPPs was also investigated. Based on the chemical parameters (e.g., surface chemistry and chemical reactivity) and the physical results (e.g., particle size and morphology), the formation mechanism of ZPPs was proposed. The UV shielding performance of polyvinyl alcohol (PVA) films containing core-shell, Pickering-like, and hollow ZPPs was evaluated by UV-visible spectroscopy.

## 2. Materials and Methods

### 2.1. Materials

Styrene (St) (Pure ≥ 99%, Sigma-Aldrich, St. Louis, MO, USA) and methyl methacrylate (MMA) (99%, Sigma-Aldrich) were purified by passing through a column pack with neutral and basic aluminum oxide (Purum, Fluka, Honeywell, NC, USA). Acrylic acid (AA) (ACS reagent, Sigma-Aldrich) was distilled before use. Divinyl benzene (DVB) (Technical grade 80%, Fluka), poly (vinyl alcohol) (PVA) (Mw = 58000–124,000, Sigma-Aldrich), potassium persulfate (KPS) (ACS 99%, Fluka), potassium hydroxide (KOH) (85%, Sigma-Aldrich), oleic acid (OA) (Pharma grade, Panreac, ITW, Glenview, IL, USA), hexadecane (HD) (99%, Sigma-Aldrich), sodium dodecyl sulfate (SDS) (98.5% GC, Sigma-Aldrich), and zinc acetate dehydrate (Zn (CH_3_COO)_2_·2H_2_O) (98%, Sigma-Aldrich) were used as received. Ethanol (95%, RCI Labscan, Bangkok, Thailand), cyclohexane (≥99.8%, Sigma-Aldrich), and deionized (DI) water were applied throughout the work.

### 2.2. Synthesis of OA-ZnO NPs

ZnO NPs were prepared by the co-precipitation method as described elsewhere [19]. Briefly, 1.75 mmol of Zn (CH_3_COO)_2_·2H_2_O (0.38 g) in ethanol (35 mL) were added into a three-necked flask fitted with a reflux condenser and mechanical stirrer at 80 °C under stirring for 2 h. Then, 1.5 mol L^−1^ KOH in an anhydrous ethanol solution (1.5 mL) was poured into the mixture (10 mL) while vigorously stirring at 70 °C for 5 min, followed by adding excessive cyclohexane. After being washed with ethanol via centrifugation thrice, OA (0.01 mL) was added into the synthesized ZnO NPs (0.4 g) suspended in ethanol (40 mL) and stirred for 24 h. Free OA was removed by centrifugation and the OA-ZnO NPs were dried in a vacuum oven at 60 °C overnight before use.

### 2.3. Synthesis and Characterization of ZPPs

ZPPs were synthesized via the mini-EP process by mixing OA-ZnO NPs (0.2 g) with St or MMA monomer (2 mL), AA (0.06 mL, 3% wt based on St), and HD (0.06 mL, 3% wt based on St) [17]. After sonicating for 2 min, SDS (1.5% wt of total volume) in an aqueous solution was added, and the mixture was stirred for 1 h before being ultrasonicated for 4 min at 60% amplitude (Branson 450 digital sonifier, Marshallscientific, Hampton, NH, USA) in an ice bath. After raising the temperature to 72 °C, the KPS solution (0.5% wt based of St) was poured into the emulsion to allow for the polymerization for 8 h under an N_2_ atmosphere. The concentrations of SDS surfactant, KPS initiator, and DVB comonomer were varied, as shown in Table 1.

The size and size distribution of NPs were measured thrice using the dynamic light scattering (DLS) technique (Malvern, Nano ZS, Malvern Panalytical Ltd., Worcestershire, UK) at 25 °C. The particle morphology was observed under transmission electron microscopy (TEM, Hitachi HT7700, Hitachi High-Tech, Tokyo, Japan) and field emission scanning electron microscopy (FE-SEM, Hitachi SU-8010, Hitachi High-Tech). The amount of ZnO NPs in ZPPs was determined by thermogravimetric analysis (TGA, TA Instruments, SDT2960 Simultaneous DTA-TGA, TA Instruments, Chicago, IL, USA), run from room temperature to 700 °C, with a heating rate of 10 °C/min. The chemical composition of hybrid particles was studied by Fourier-transform infrared spectroscopy (FTIR, Spectrum GX, Perkin Elmer, Waltham, MA, USA), whereas their crystallinity was investigated by using the X-Ray diffraction technique (XRD, D8 Venture, Bruker, Billerica, MA, USA).

### 2.4. UV Shielding Performance of PVA and Composite Films

The coating suspensions were prepared by a mixing powder of OA-ZnO NPs or ZPPs (0.03 g, 1%wt of total volume) with a 6% PVA solution (3 mL), using magnetic stirrer, for 4 h. The suspension mixture (wet film thickness of 100 μm) was cast onto the glass slide (22 × 22 mm^2^) using a laboratory film applicator and allowed to dry under ambient conditions. The absorption and transmittance of the pristine PVA and composite films on the glass slides was determined using UV-visible spectrophotometer (UV-2600 Shimadzu, Kyoto, Japan) thrice. The scanning rate was 190 nm min^−1^.

## 3. Results

### 3.1. Analysis of OA-ZnO NPs

TEM and SEM images of the prepared ZnO NPs in Figure S1A,B show the spherical particles with an average size of 11.8 ± 1.8 nm. However, large aggregates of as-made ZnO NPs were observed in both micrographs, possibly caused by the hydrophilic interaction among hydroxyl groups on the particle surface [7,20]. The XRD pattern in Figure S1C displays the well-defined peaks at 31.7°, 34.4°, 36.2°, 47.4°, 56.5°, 62.8°, 66.0°, 67.9°, and 69.1°, corresponding to the diffractions of (100), (002), (101), (102), (110), (103), (200), (112), and (201) planes of ZnO, respectively. The good agreement between the standard diffraction peaks and the obtained peaks indicated the successful preparation of the ZnO in nanoscale [21]. The high purity of the synthesized product was confirmed by the similar standard pattern. Moreover, this diffraction pattern indicated the good crystalline nature in the hexagonal structure of resulting materials [2].

**Figure 1:**
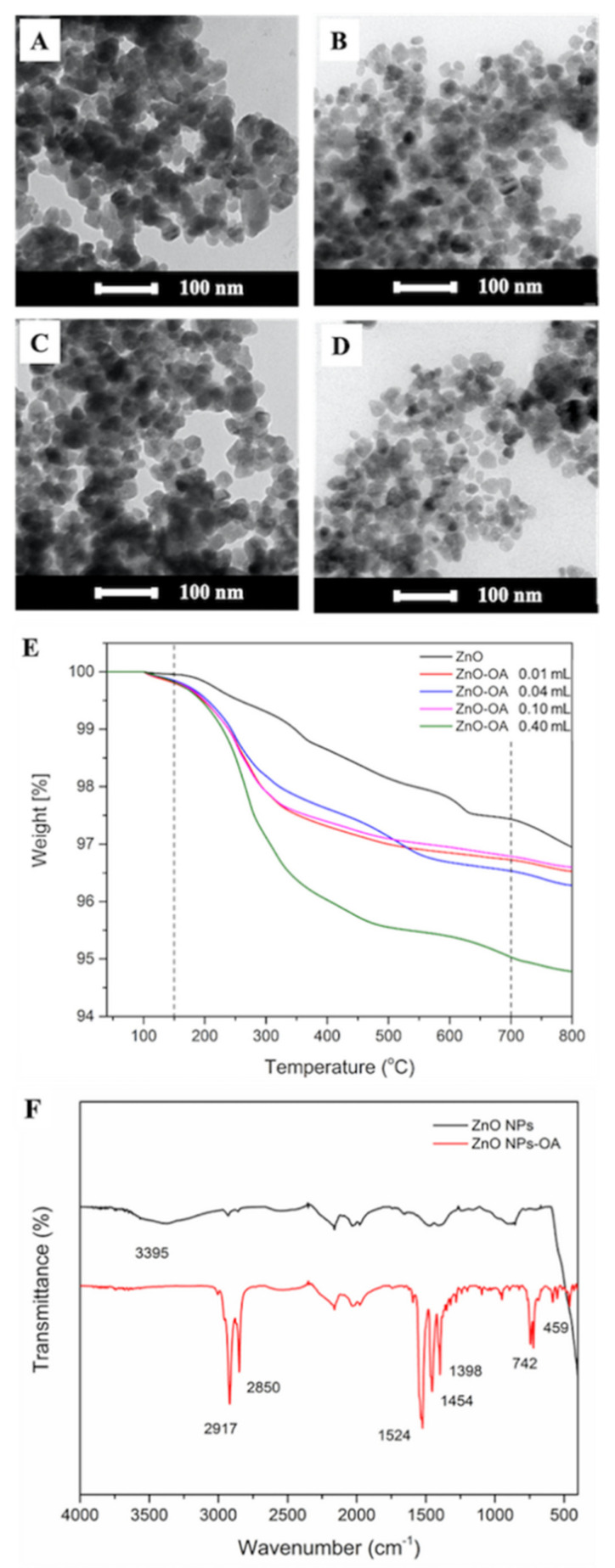
TEM images (**A**–**D**), TGA thermogram (**E**) of OA-ZnO NPs using the added OA content of 0.01, 0.04, 0.10, and 0.4 mL, and (**F**) FTIR spectra of bare ZnO and OA-ZnO NPs (4.8 wt% OA).

To reduce the agglomeration of ZnO NPs, and to improve its dispersibility in monomer droplet, the particle surface was modified with the commonly used coupling agent, i.e., OA. TEM images of OA-ZnO NPs, having various OA contents, are shown in Figure 1A–D. The better dispersion of ZnO NPs was obtained when the added OA was higher than 0.01 mL. The adsorbed OA broke up the agglomerates by decreasing their surface energy and by increasing steric repulsion [22]. TGA thermograms in Figure 1E confirmed the existence of OA in the synthesized OA-ZnO NPs. The weight loss of the bare and the four samples of OA-ZnO NPs with the added OA of 0.01, 0.04, 0.10, and 0.40 mL, in the range of 150–700 °C, were 2.5, 3.1, 3.3, 3.0, and 4.8%, respectively. The high decomposition rate of OA-ZnO NPs with 0.40 mL OA (0.0004 mg/min) in Figure S2 further confirmed the high amount of OA on ZnO NPs surface compared to bared ZnO NPs and the other OA-ZnO NPs (0.0002 mg/min). The rapid weight loss at ca. 350 °C was due to the decomposition of adsorbed OA molecules [23]. Due to the good NPs distribution in Figure 1D and the high amount of chemically adsorbed OA from the TGA, an OA of 4.8 wt% (determined from the TGA) was chosen for preparing the OA-ZnO NPs used in further studies.

The FTIR spectra of bare ZnO and OA-ZnO NPs with 4.8 wt% OA are shown in Figure 1F. Compared to the peak at 3395 cm^−1^ corresponding to O–H stretching of bare ZnO NPs [1], the sharp peaks at 2917 and 2850 cm^−1^ in the spectrum of OA-ZnO NPs, corresponding to C–H stretching vibrations, and those at 1398 and 1454 cm^−1^, relating to C–H bending vibrations [22], implied the presence of OA. The absence of O–H stretching after modification and the C=O stretching of OA indicated the interaction between the O–H group on the ZnO surface and the carboxylate moiety of OA. The corona of the hydrocarbon chain as the outermost layer [10,23] resulted in the good particle stability due to the steric effect and agreed well with the image observed in Figure 1D. The lower intensity of the ZnO characteristic peak at 459 cm^−1^, compared to that of bare ZnO NPs, also supported the successful surface modification by OA [13].

### 3.2. Analysis of ZPPs

#### 3.2.1. Effect of Surfactant Concentration

**Figure 2:**
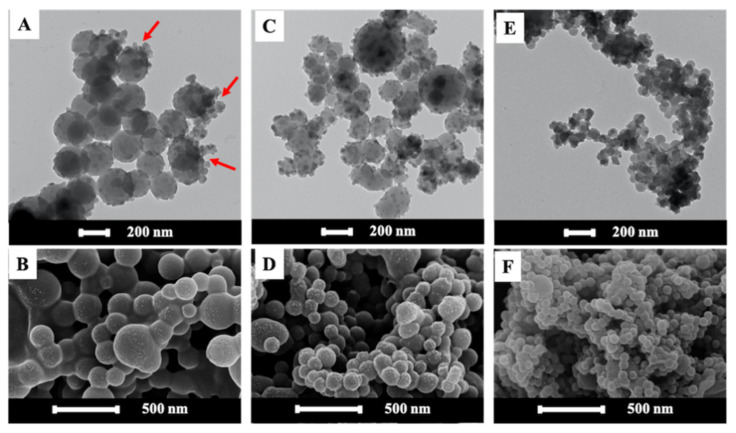
TEM and SEM images of spherical ZPPs prepared from P(St/AA) with various SDS concentrations of 0.5 (below CMC) (**A**,**B**), 1.5 (at CMC) (**C**,**D**), and 3.0% (above CMC) (**E**,**F**).

Aside from the time and amplitude of the sonication, the amount of surfactant usually influences the equilibrium rate, size, and stability of nanodroplets, or miniemulsion during ultrasonicating, as well as particle morphology [24,25]. Higher surfactant concentrations result in lower surface tension and, hence, the smaller latex particle size [26]. The effect of the concentration of SDS on the size of the ZPPs, made from P(St/AA) synthesized with 0.5%KPS, was studied. Table 1 shows that the average particle size decreased from 200.5 ± 24.8 to 139.8 ± 41.1 and 64.5 ± 4.3 nm, when increasing SDS from 0.5 (below the CMC), 1.5 (at the CMC) to 3.0% (above the CMC). This agreed well with the previous works reporting that, at above the CMC, there were enough SDS molecules for stabilizing a large number of small monomer droplets formed after shearing [26]. The SDS concentration also affected the morphology of the composite particle. Figure 2 shows the TEM and SEM images of the spherical ZPPs prepared from 0.5 (Figure 2A,B), 1.5 (Figure 2C,D), and 3.0% (Figure 2E,F) of SDS using PS as the matrix.

The polydispersed spherical ZPPs of P(St/AA), containing a high amount of ZnO NPs, were observed at all conditions. At below or equal to the CMC of SDS, Figure 2A–D revealed the Pickering-like morphology, i.e., small OA-ZnO NPs (dark sphere), located on the surface of the bigger PS core (lighter sphere). The OA-ZnO NPs, having higher hydrophilicity than PS, were separated and pushed towards the aqueous phase and played the role as another stabilizer of composite particles. If there was enough available space at the particle surface, i.e., at low SDS in Figure 2A,B, ZnO NPs could embed more at the PS and water interface. More SDS at its CMC, in Figure 2C,D, might repulse non-polar OA, resulting in the protrusion of ZnO NPs from the PS phase. At above the CMC, the appearance of ZnO NPs as a Pickering stabilizer on the small-sized ZPPs core was not clearly observed in Figure 2E,F. This might be due to the higher surface area of smaller PS core particle, which allowed for the faster trapping of oligoradicals and, hence, the higher polymerization rate, especially at the surface of miniemulsion. This rationale agrees well with the following Equation (1):

(1) $$r_{p\;}=\frac{K_{p}.{\left[M\right]}_{p}.\bar{n}.N_{p}}{N_{AV}}$$

where $r_{p\;}$ is the rate of polymerization, $K_{p}$ is propagation rate coefficient and ${\left[M\right]}_{p}$ is the monomer concentration in the particle, while $\bar{n}$, $N_{p}$, and $N_{AV}$ are the average number of the radical, the number of the particle, and Avogadro’s number, respectively [27]. Consequently, the high viscosity in each nanodroplet impeded the migration of OA-ZnO NPs towards the particle surface, whereas a large amount of the SDS molecules rapidly moved to cover the particle surface [28]. This effect is more pronounced in the case of the small-sized and oligomer-rich monomer droplet. The complete encapsulation of OA-ZnO NPs within the PS matrix in Figure 2E,F depicts a kinetically controlled morphology [25]. The existence of OA-ZnO NPs on the ZPPs surface decreased the absolute zeta potentials, i.e., from −54.7 ± 3.3 mV to −26.1 ± 1.1, −24.8 ± 0.6, and −32.3 ± 2.3 mV when using 0.5, 1.5, and 3.0% SDS, respectively [29]. This implied that the combined electrostatic repulsion, from ionic groups of SDS/KPS/AA, and the steric repulsion, from hydrocarbon chains of OA on ZnO, were responsible for the high stability of ZPPs at all SDS concentrations.

The FTIR spectra in Figure S3A show the characteristic peaks of the benzene ring in the St at 3027 cm^−1^ for C=C–H stretching, 1584 and 1493 cm^−1^ for C–C stretching, 757 and 697 cm^−1^ for C–H bending [13]. C–H bending (1452 cm^−1^) and Zn–O bending (539 cm^−1^) corresponded to OA-ZnO NPs on the PS surface. The TGA thermogram of ZPPs in Figure S3B confirmed the decomposition of the organic PS ranging from 350–500 °C [30]. The % weight residue of the ZnO NPs were 26.7, 12.8, and 15.3% for the ZPPs using 0.5, 1.5, and 3.0% SDS, respectively. At below the CMC, the extent of polymerization at the surface of the nano-sized monomer droplets, caused by diffused oligoradicals, might be low, and the migration of OA-ZnO NPs would not be hindered. It was then observed that there were clusters of ZnO NPs on the surface of PS (see indicated arrows in Figure 2A). High aggregation of ZnO NPs at the composites interface below the CMC related well with high value of % weight residues. Considering the morphology, the average particle size distribution, zeta potential, and inorganic content, the suitable content of SDS providing stabilized composite particles at a CMC of 1.5% was chosen for the further experiments.

#### 3.2.2. Effect of Initiator Content

Initiator also exerts an influence on the particle size and morphology of composite particles generated by the mini-EP. Table 1 shows the average size of ZPPs, which increased from 139.8 ± 41.1, 170.6 ± 21.0, to 174.3 ± 41.2 nm, when increasing the amount of water soluble initiator, KPS, from 0.5, 1.0, to 2.0%, respectively. When the higher concentration of KPS was added, the larger number of short chain oligoradicals, rapidly formed, would stay in water for a longer period due to their higher hydrophilicity. Until reaching its critical chain length, these oligoradicals were adsorbed on the surface of nanodroplets and continued polymerization [18]. This period allowed the coalescence of the small miniemulsion droplets and, hence, the formation of larger composite particles. The process of an increase in primary radical concentration, which promotes the particle coagulation, could be expressed by the modified Von Smoluchowski Equation (2) [31,32]:

(2) $$B\left(i,j\right)=f\left(\gamma \right)N\left(i\right)N\left(j\right){\left[d_{p}\left(i\right)+d_{p}\left(j\right)\right]}^{3}$$

where $B\left(i,j\right)$ are the number of collisions between particles class *i* and *j*, and $f\left(\gamma \right)$ is constant. *N*(*i*) and *N*(*j*) are the number of particles in class *i* and *j*, whereas $d_{p}\left(i\right)$ and $d_{p}\left(j\right)$ are particle diameter of class *i* and *j*, respectively. *B*(*i*,*j*) and, consequently, the extent of the coagulation directly increased with the increasing the number of primary particles of St at the initiation step, leading to the agglomeration between primary particles and monomer-swollen micelles. The number of primary particles and miniemulsion droplets (*N*(*i*) and *N*(*j*)) were depleted during polymerization, whereas the size of monomer-swollen latex particles increased. A similar observation was reported in the emulsion polymerization system of St when methanol was used as co-solvent, i.e., the final size of the PS latex particles increased with the amount of KPS, due to the particle coalescence during polymerization [32]. Increasing the amount of initiator enhanced the extent of the particle coagulation. In addition, as a water soluble initiator and inorganic salt, dissolution and decomposition of KPS enhanced the ionic strength of the aqueous phase and, consequently, suppressed the charge of existing species, e.g., primary particles and monomer-swollen micelles, leading to the particle coagulation [32].

**Figure 3:**
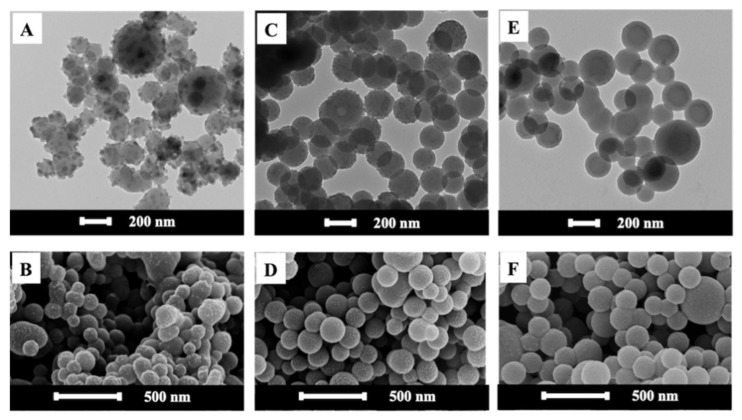
TEM and SEM images of spherical ZPPs synthesized by using 0.5 (**A**,**B**), 1.0 (**C**,**D**), and 2.0% (**E**,**F**) of KPS initiator.

The amount of KPS also affected the morphology of ZPPs as presented in Figure 3. The TEM images of ZPPs using 0.5 and 1% KPS in Figure 3A,C reveal a spherical PS core surrounded with small OA-ZnO NPs in Pickering-like or raspberry-like structure, which correlated well with their SEM images in Figure 3B,D. As OA on ZnO NPs have a higher affinity with oligoradicals, as compared to St monomer and PS, OA-ZnO NPs moved outwards on the surface of the monomer-swollen micelles [18]. In the case of 2% KPS, both images of ZPPs in Figure 3E,F show a relatively smooth surface with few portions of attached ZnO NPs. The decrease of the protrusion of ZnO NPs on the ZPP surface might be attributed to the high polymerization rate. The large amount of St oligoradicals rapidly polymerized, and consequently, the polymer impeded the migration of OA-ZnO NPs from the PS matrix as already mentioned. This confirmed that the morphology transitioning from the non-Pickering (at 2% KPS) to Pickering-like (at 0.5–1% KPS) was kinetically controlled. The negative zeta potentials of all ZPPs derived from the sulfate groups of SDS, KPS, and carboxyl groups of OA-ZnO NPs/AA, which confirm the good dispersion of ZPPs in an aqueous solution.

The FTIR spectra of ZPPs prepared using different concentrations of KPS are shown in Figure S4A. Although similar FTIR spectra were observed, it is worth noting that the intensity of C-H bending (1452 cm^−1^) and Zn–O bending related to the existence and location of the OA-ZnO NPs in the ZPPs. The % weight residues of ZPPs from the TGA thermograms in Figure S4B of 12.8, 12.2, and 8.9 for 0.5, 1.0, and 2.0% KPS, respectively, agreed with the TEM images of the ZnO NPs on the ZPPs in Figure 3.

#### 3.2.3. Effect of Crosslinking Agent

**Figure 4:**
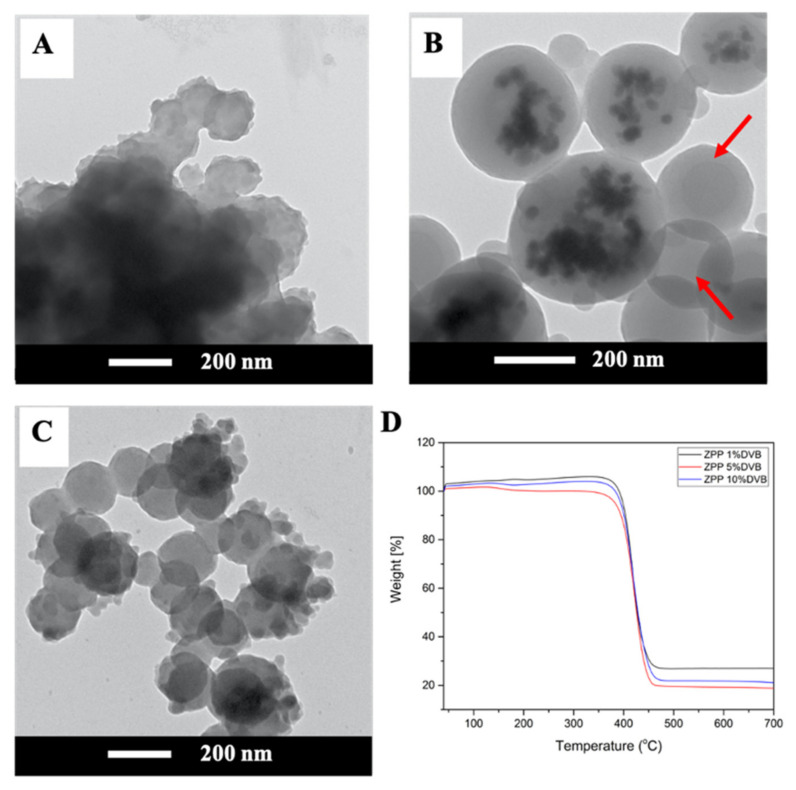
TEM images of spherical ZPPs by varying DVB from (**A**) 1, (**B**) 5, and (**C**) 10%. and (**D**) the TGA thermograms.

To further validate the contribution of kinetic parameters on the morphology of ZPPs, the influence of the crosslinking agent, DVB, was investigated. Having two reactive sites, the crosslinking agent accelerates the polymerization rate and viscosity in the growing monomer-swollen micelles [33,34]. The TEM images in Figure 4A–C reveal the morphologies of the ZPPs containing ZnO NPs in PS made from 1, 5, and 10% DVB. When the amount of DVB increased, the resulting ZPPs changed from ZnO NPs coated on PS core or Pickering-like morphology (1% DVB) to ZnO NPs embedded in the PS particle (5% DVB) and, finally, to the PS particle inhomogeneously decorated with ZnO NPs on the surface. At the same time, average particle sizes varied from 182 ± 49.9 to 340.0 ± 48.3 and 199.0 ± 67.2 nm. At 1% DVB, the morphology of ZPPs was relatively similar to the non-crosslinked ZPPs (Figure 2C), i.e., Pickering-like morphology, but the protrusion of the ZnO NPs seemed to be lower. This is because the presence of DVB promotes the formation of primary particles at the beginning of polymerization [34,35]. The larger particle size and the entrapment of ZnO NPs in ZPP when using 5% DVB were due to the fact that DVB raises the internal viscosity and the crosslinked density in growing particles, which could then trap ZnO NPs before exerting to the surface of the ZPP. However, some ZnO-free particles (see arrow) were found and their sizes (256.4 ± 6.7 nm) were smaller than that of the composites (340.0 ± 48.3 nm) because of the inhomogeneous distribution of ZnO NPs and the non-uniformity during polymerization. At 5%, DVB might not be completely miscible with St, since solubility of DVB (δ = 8.5 (cal/cm^3^)^1/2^) is lower than that of St (δ = 9.85 (cal/cm^3^)^1/2^) [19]. In addition, OA-ZnO NPs (δ = 8.2 (cal/cm^3^)^1/2^) might have higher affinity to DVB or the DVB-rich phase than St. High crosslinker, at 10% DVB, causes surface shrinkage, which subsequently yields smaller particle size [36]. The increased DVB could cooperate with the HD (δ = 8.0 (cal/cm^3^)^1/2^) hydrophobe to suppress the Ostwald ripening process, which also results in the formation of small particles. Consequently, the ZnO NPs could be protruded to the surface of the particle. ZnO-free particles were also found, as in the case of 5% DVB.

Zeta potential and % weight residue of ZPPs prepared at different DVB percentages are presented in Table 1. All crosslinked ZPPs had lower absolute zeta potential values compared to the non-crosslinked ones [37]. Due to the higher polymerization rate, DVB residing inside the monomer-swollen micelles might pull the diffused oligomer and OA-ZnO NPs from the surface of miniemulsion to polymerize, and the carboxylic end of OA modified ZnO surface is then trapped in the inner part of the emulsion.

The composition of ZPPs was confirmed by TGA thermograms in Figure 4D. The weight residues of crosslinked ZPPs were 26.9, 19.5, and 21.9 for 1, 5, and 10% DVB, respectively, and were significantly higher than that of the non-crosslinked ZPPs (12.8%). The higher ZnO content are attributed to the higher polymerization rate caused by DVB (reactivity ratios of DVB = 1.3 and of St = 0.55) [38] and the miscibility between OA-ZnO and DVB, as already mentioned.

**Figure 5:**
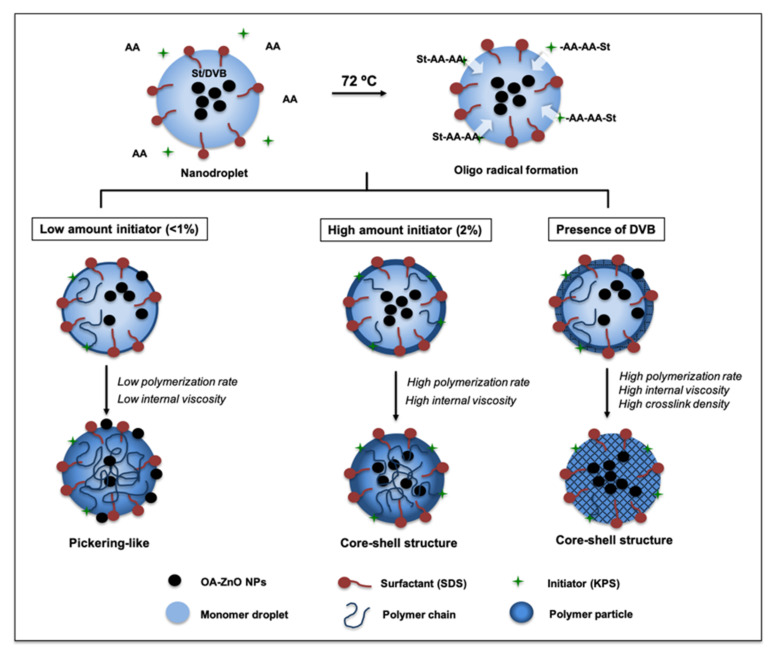
Proposed mechanism for the formation of Pickering-like and spherical ZPP.

Figure 5 presents the proposed formation mechanism of ZPPs. OA-ZnO NPs are included in the SDS-stabilized monomer droplets. Aside from St (and DVB), a small amount of AA monomer existed in the reaction system and the polymerization of St/AA was initiated in an aqueous phase [18] by KPS to form radical species, which then continued polymerizing in the nanodroplet. Due to the low number of radical species compared with the amount of initiator, the polymerization rate in the emulsion droplet was low. Upon polymerization, chemical affinity between the OA-ZnO NPs and the polymer chains became lower. OA-ZnO NPs can be trapped or pushed away from the polymer chains. The morphology or resulting ZPPs was dependent on the interfacial tension between OA-ZnO NPs and the synthesis of the parameters/surroundings and polymerization rate in the nano-size emulsions [25]. In the absence of DVB, at a low concentration of KPS (≤1%) and SDS (≤1.5%), the polymerization rate is relatively low, and so too is the internal viscosity. ZnO NPs, which are more hydrophilic than the growing polymer chain, can be pushed outwards and Pickering-like or ZnO-coated PS are then formed. This morphology can be considered as a thermodynamically driven structure. At higher concentrations of KPS (2.0%), the larger number of oligoradical generated cause an increase in polymerization rate and, hence, internal viscosity. The migration of ZnO NPs to the water and polymer interface is impeded. The core and shell, or sparingly protruded ZnO NPs, are then observed. These effects are more pronounced when DVB, crosslinker, is present in the system [17]. Our results also illustrate that crosslinker significantly affects the morphology of the resultant composites. The high internal viscosity and crosslink density caused from polymerization of DVB and St keep the ZnO NPs from moving outward. The ZnO core/PS shell structure is formed as a result of the kinetically driven system.

#### 3.2.4. Effect of Monomer Hydrophilicity

**Figure 6:**
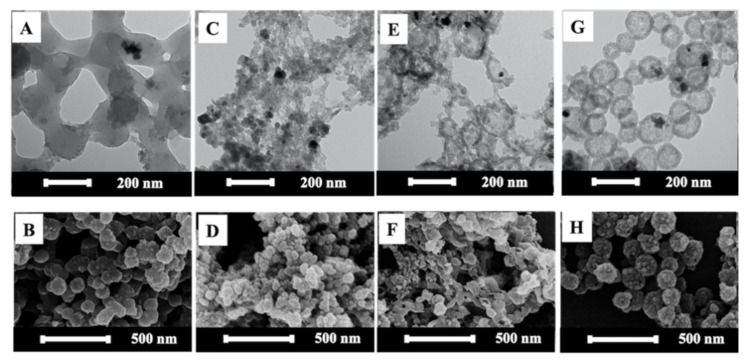
TEM and SEM images of spherical ZPP using MMA as a main monomer and varying DVB crosslinking agent from 0% (**A**,**B**), 1.0%(**C**,**D**), 5.0% (**E**,**F**), and 10.0% (**G**,**H**).

As previously discussed, on the interfacial tension and kinetic effects on composite morphology, St was substituted with the more hydrophilic monomer, MMA, with a similar reactivity ratio of 0.58 [39]. The effect of the DVB content, i.e., 0, 1, 5, to 10% wt of total monomers, on the morphology of the ZPPs and the position of the ZnO NPs in the composite particle was investigated under TEM and SEM, as presented in Figure 6. Without DVB, the aggregates of the spherical ZPPs with OA-ZnO NPs on the surface or a polymer core/ZnO shell appeared in Figure 6A,B. This was due to the incompatibility between MMA (δ = 11.67) and OA-ZnO NPs (δ = 8.2). To lower interfacial tension between MMA and OA-ZnO NPs, OA-ZnO NPs tended to agglomerate and separate from MMA during polymerization.

Once DVB was introduced, the morphologies of the resulting ZPPs were altered from non-hollow (0% DVB) to clusters of small composites (1% DVB), the mixture of small composites and hollow particles (5% DVB), and finally well-defined hollow ZPPs (10% DVB). Despite being present at a low concentration (1%), DVB strongly disturbed the formation of composites, as indicated by the aggregation of small particles (Figure 6C,D). Due to the large difference in solubility between MMA and DVB, MMA can dissolve both in an emulsion droplet and in an aqueous phase, while DVB can only reside in an emulsion droplet HD and ZnO NPs [18]. Oligoradicals react with DVB/MMA at the surface of emulsion droplets. Interestingly, the size of crosslinked ZPPs (1%, 32.0 ± 6.8 nm) is much smaller than non-crosslinked ZPPs (150.7 ± 16.5 nm). This might imply that the non-uniform polymerization occurs at the water and monomer droplet interface, which may cause the osmosis of water into the droplet to compensate for the depletion of monomers, especially MMA. Having an inadequate amount of crosslinker means not being able to hold the particles intact and, consequently, large composite particles burst into smaller particle sizes.

In Figure 6E,F, when using 5% DVB, the mixture of hollow particles (120 ± 25.8 nm) and aggregates of small particles, similarly found in the 1% DVB sample, were observed. It is worth noting that the size of the small particles in the aggregates was similar to the shell thickness of hollow particles, i.e., ca. 30 nm. This result supported our aforementioned assumption. Owing to the low miscibility between the relatively hydrophilic MMA and the hydrophobic DVB and the use of water-soluble initiator KPS, MMA migrated towards the water and emulsion interface to copolymerize, which led to the formation of a void or polymer-free core [40]. If crosslinked density of the shell was low, it would not have enough rigidity to maintain the whole particle. As previously reported, the type and amount of crosslinker affected the surface morphology and particle size of hollow and non-hollow polymeric particles [18,41]. The surface roughness of HoMPs increased with the increasing the amount of DVB [18]. When using ethylene glycol dimethacrylate (EGDMA) crosslinker (10–90 mol%), pores on the surface of non-hollow PMMA particles were formed. The higher the concentration of EGDMA, the bigger the pore size. The presence of a pore was the result of a microphase separation during polymerization between MMA and EGDMA [41]. In our case, the heterogeneity should be more pronounced due to the large solubility difference between MMA and DVB.

With increasing the DVB concentration to 10% (Figure 6G,H), hollow ZPPs with complete shell (thickness of 35 nm) were observed and small particles with ca. 30 nm were barely seen. Figure 6H clearly depicted the rugged surface of hollow composites, where OA-ZnO NPs were trapped in the cavity. If the location of OA-ZnO NPs was considered as a tracer of reaction rate, the presence of OA-ZnO NPs inside the hollow ZPPs suggested the fastest rate of polymerization. The existence of a sufficient amount of DVB in the reaction system accelerated the polymerization and crosslinking. The resulting particles had highly a crosslinked network that was robust enough to hold the particle shape [34]. This collective evidence supported the assumptions that the bursting of lightly crosslinked shells generated small particles and aggregates. It emphasized the kinetically governed morphology of ZPP by way of the rate of polymerization and crosslinking.

**Figure 7:**
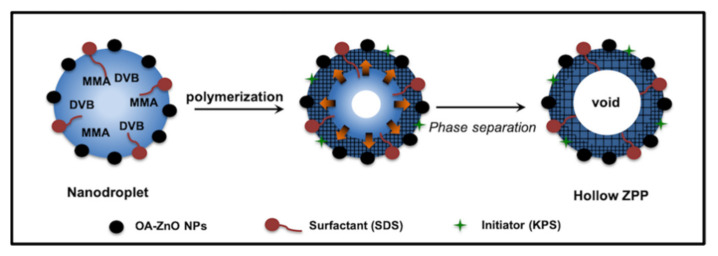
Scheme of hollow structure formation.

The formation of hollow ZPP is proposed in Figure 7 and is different from the St/DVB system. Due to its relatively hydrophilic behavior, MMA resided nearly in the interface of water and emulsion droplet [18,42], while OA-ZnO NPs, DVB, and HD were in the inner region. Thus, there was a chemical gradient in the emulsion droplet. After reaching the emulsion droplets, oligoradicals, initiated in the aqueous phase, polymerized with MMA/DVB near the surface of the emulsion droplet. DVB caused the shrinkage of the polymer shell, as seen in rugged and porous surface. Consumption of hydrophilic MMA, during polymerization, caused the osmosis of water from the continuous phase to the emulsion droplet through the pores. The degree of crosslinking took part in the physical stability of the hollow ZPP. The incorporation of ZnO NPs in the hollow ZPP composites was confirmed by the TGA analysis in Figure S5. The weight loss at 300–500 °C was 30.1, 31.2, and 57.2% corresponded to the decomposition of the organic polymer part of the PMMA-ZPPs with 1, 5, and 10% DVB, respectively. All MMA-based ZPPs showed much higher weight residue, which corresponded to the amount of ZnO NPs, than St-based ZPPs.

### 3.3. Effect of ZPP Morphology on UV Shielding Protection

**Figure 8:**
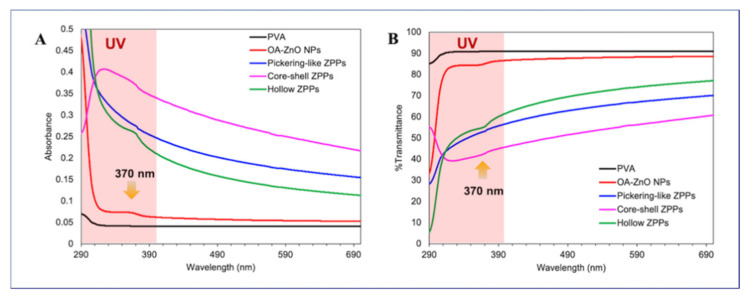
UV-visible absorption (**A**) and % transmittance (**B**) spectra in UV-visible range (300–700 nm) of Pickering-like, core-shell, and hollow ZPPs compared to OA-ZnO NPs.

To evaluate the UV-blocking of ZPPs in the coatings, ZPPs were mixed with a PVA solution prior to casting on the glass slide substrates. Although all hybrid films were prepared from mixtures containing the same solid content, the amount of ZnO NPs were different. The OA-ZnO NPs-PVA film was as transparent as the pristine PVA film on a black background (Figure S6) because the particle size of OA-ZnO NPs was small (11.8 ± 1.8 nm) and the PVA can partially form a hydrophobic interaction [43]. The hybrid films containing ZPPs were less transparent or opaque on black background and appeared transparent on white background. These results posed the influence of particle size on light scattering on different backgrounds and their visible transparency. The attenuation of light in materials is a combination of absorption and the scattering of UV and visible radiation, of which the latter causes opacity [44]. Compared to pristine films, OA-ZnO NPs-PVA film showed slightly higher absorbance over the UV-visible region, where the characteristic peak of ZnO NPs at 370 nm were observed, as presented in Figure 8A [2,23]. All the ZPPs-based PVA films presented significant enhancement of absorption over the UV and visible region than the pristine and OA-ZnO NP-PVA films, suggesting good UV and visible shielding [45]. Besides characteristic peaks of ZnO NPs, all the absorption spectra of the ZPP-based PVA films showed the scattering patterns over UV-visible region due to their large particle sizes. Hybrid PVA films containing core-shell ZPPs exhibited the highest absorbance with λ_max_ at 329 nm, followed by Pickering-like and hollow ZPPs-PVA films, respectively. The collaborating influence between the particle size of ZPPs and the location of ZnO NPs seemed to outweigh the amount of ZnO NPs in ZPPs. ZnO NPs in the core-shell ZPP were confined in the core area (about 100 nm) and coated with a PS shell, with a refractive index (1.5865) close to PVA (1.4770). These nano-size semiconductors are in close proximity to each other, which facilitated the motion of the electron in the conducting band (CB) and the hole in valence band (VB) between adjacent ZnO NPs and performed high UV absorption, as illustrated in Figure S7 [46,47]. It might be said that absorption mode plays a key role in the UV shielding of core-shell ZPPs. Aside from particle sizes and the location of ZnO NPs laying on the surface or in the shell of ZPPs, the absorbance and absorption patterns of Pickering-like and hollow ZPPs are comparable. Multiple light scattering, between the high refractive index of ZnO NPs (1.9596) at the interface and the low refractive index of the PS core for Pickering-like ZPPs or the air void (1.0003) for hollow ZPPs, is possibly the major contributor for their shielding properties [48].

Similar results were observed in the transmission spectra as shown in Figure 8B. Transmittance of pristine and OA-ZnO NPs-PVA films were, respectively, nearly 90% and 83%, which agreed well with the optical images in Figure S6. The transmittance of ZPPs-PVA films were lower than the pristine and OA-ZnO NPs-PVA films, in which core-shell ZPPs-PVA films presented the lowest transmittance value among ZPPs, followed by Pickering-like and hollow ZPPs-based PVA films. Despite exhibiting some degree of opacity, all ZPPs-based PVA films showed ca. 45–65% transmittance in the UV region and ca. 50–80% in the visible region. The strong UV absorption and light transmittance in the visible range suggested the advantages of the encapsulation of ZnO NPs in particulate form, making them a potential candidate for UV-shielding applications.

## 4. Conclusions

The morphologies of ZPPs of P(St/AA), prepared by mini-EP and varying from Pickering-like to core-shell, were kinetically controlled by adjusting the polymerization factors, i.e., the amount of SDS, KPS, and DVB. When increasing the SDS concentration (0.5–3.0%), the size of the ZPPs decreased from 200.5 ± 74.8 nm to 64.5 ± 4.3 nm. The Pickering-like ZPPs were obtained at below and equal to its CMC. At above the CMC, the complete encapsulation of the OA-ZnO NPs within the ZPPs depicted a kinetically controlled morphology due to the higher polymerization rate and, hence, higher viscosity in miniemulsion. The amount of KPS influenced the location of the OA-ZnO NPs on the ZPPs, i.e., the transition from the non-Pickering (at 2% KPS) to Pickering-like (at 0.5–1% KPS), was kinetically controlled. Incorporation of DVB further validated the kinetic contribution on the morphology. The acceleration of polymerization rate and high viscosity of DVB caused the entrapment of OA-ZnO NPs within the polymer particle. The ZPPs morphology changed from Pickering-like (1% DVB) to PS-coated ZnO NPs (5% DVB) and, finally, to PS inhomogeneously decorated with ZnO NPs (10% DVB) on the surface. For the further study of interfacial tension and kinetic effect, St was replaced by MMA. The sufficient amount of 10% DVB provided not only the high crosslinked density, but also the stable hollow ZPPs. In addition, all ZPP-incorporated PVA films showed superior UV shielding performance in comparison with pristine PVA and ZnO NP-incorporated PVA films. The large amount of ZnO NPs located in core were responsible for the high absorption of core-shell ZPPs, while the difference in refractive index between ZnO NPs on the interface and the polymer/void core caused the multiple light scattering as a dominant mode of shielding for hollow and Pickering-like ZPPs. All PVA films containing ZPPs also showed relatively high transparency. Therefore, understanding reaction kinetics attributed from polymerization parameters allows for the design of the morphology of inorganic polymeric composites to meet specific needs.

## Figures and Tables

**Table 1:** Formula of ZPPs with relevant characteristics, i.e., average particle size from TEM, zeta potential, and %weight residue from TGA.

System	Composition				Particle Size (nm)	Zeta Potential (mV)	%Weight Residue from TGA	Morphology
	Monomer	DVB	KPS	SDS				
1	St	-	0.5%	0.5%	200.5 ± 24.8	−26.1 ± 1.1	26.7	Pickering-like
2	St	-	0.5%	1.5%	139.8 ± 41.1	−24.8 ± 0.6	12.8	Pickering-like
3	St	-	0.5%	3.0%	64.6 ± 4.3	−32.3 ± 2.3	15.4	Pickering-like
4	St	-	1%	1.5%	170.6 ± 21.0	−28.2 ± 0.5	12.2	Pickering-like
5	St	-	2%	1.5%	174.3 ± 41.2	−26.7 ± 0.2	8.9	Pickering-like
6	St	1%	0.5%	1.5%	182.3 ± 49.9	−12.2 ± 5.3	26.9	Pickering-like
7	St	5%	0.5%	1.5%	340.0 ± 48.3	−17.6 ± 4.1	19.5	Core-shell
8	St	10%	0.5%	1.5%	192.0 ± 67.2	−18.7 ± 5.4	21.9	Pickering-like
9	MMA	1%	0.5%	1.5%	32.0 ± 6.8	−23.2 ± 0.5	69.9	-
10	MMA	5%	0.5%	1.5%	118.9 ± 25.8	−22.3 ± 0.5	68.8	Hollow
11	MMA	10%	0.5%	1.5%	119.7 ± 28.5	−20.9 ± 0.8	42.8	Hollow

## Data Availability

The data presented in this study are available on request from the corresponding author.

## Supplementary Materials

The following are available online at https://www.mdpi.com/article/10.3390/polym13152526/s1, Figure S1: TEM (A) and SEM (B) images and XRD pattern (C) of ZnO NPs, Figure S2: Differential TGA diagram of bare ZnO NPs and OA-ZnO NPs using the added OA content of 0.01, 0.04, 0.10, and 0.4 mL, Figure S3: (A) FTIR spectra and (B) TGA thermograms of ZPPs prepared by varying the amount of SDS, i.e., 0.5 (black), 1.5 (red), and 3.0% (blue), Figure S4: (A) FTIR spectra and (B) TGA thermograms of ZPPs prepared by varying the amount of KPS (0.5, 1.0, and 2.0%), Figure S5: TGA thermograms of ZPPs using PMMA mixed with 1, 5, and 10% DVB, Figure S6: Photographs of PVA film and PVA films containing 1 wt% of OA-ZnO NPs, core-shell ZPPs, Pickering-like ZPPs, and hollow ZPPs coated on glass slide placed on black and white backgrounds, and Figure S7: Schematic diagram of light absorption and multi light scattering of core-shell, Pickering-like and hollow ZPPs.
