# Supplementary material for: Facile Control of Structured ZnO Polymeric Nanoparticles through Miniemulsion Polymerization: Kinetic and UV Shielding Effects
Source: Polymers (Basel). 2021 Jul 30;13(15):2526. doi: 10.3390/polym13152526 (PMC8347249; doi:10.3390/polym13152526)
Supplement: Supplementary file 1 [file polymers-13-02526-s001.zip › polymers-1283271-supplementary.pdf]

Supporting information

Facile Control of Structured ZnO Polymeric Nanoparticles through Miniemulsion Polymerization: Kinetic and UV Shielding Effects

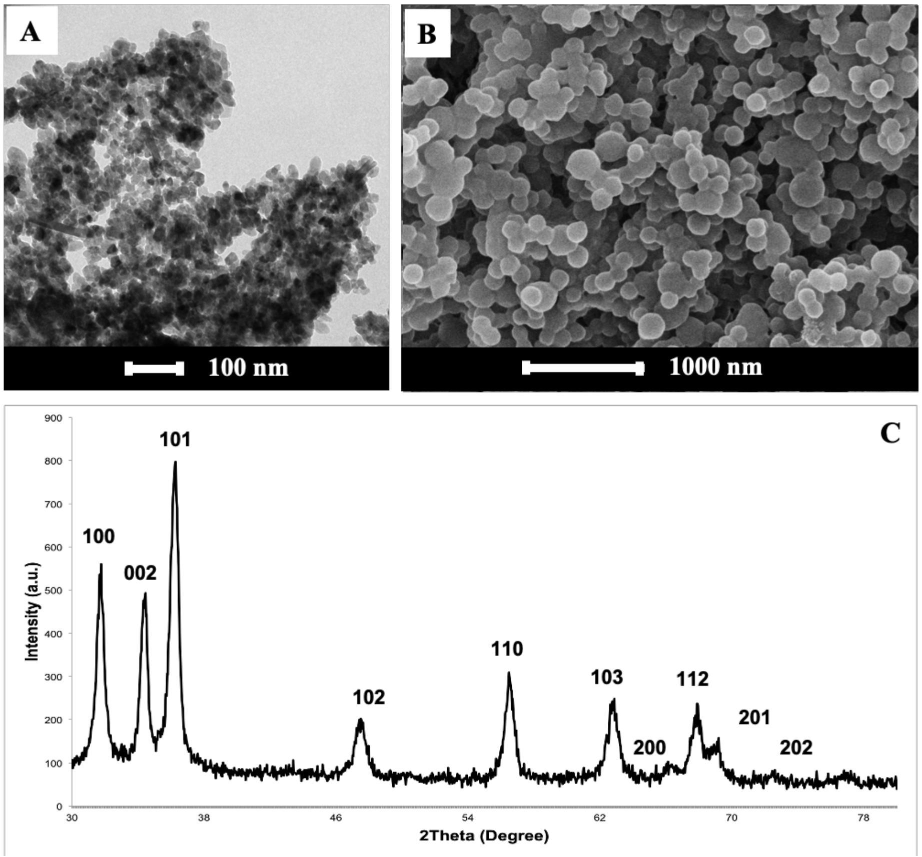

Figure S1. TEM (A) and SEM (B) images and XRD pattern (C) of ZnO NPs.

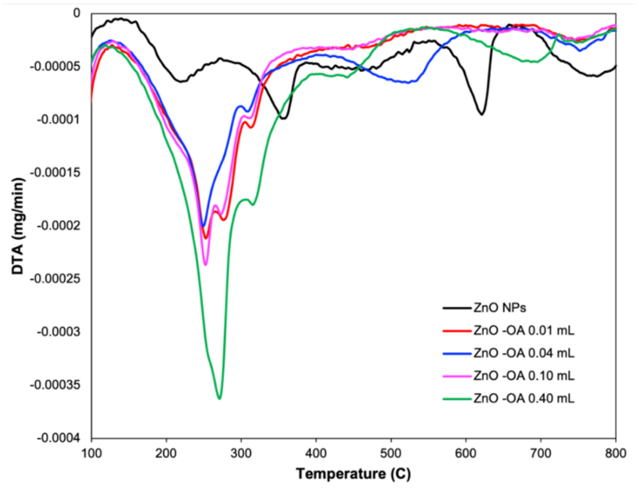

Figure S2. Differential TGA diagram of bare ZnO NPs and OA-ZnO NPs using the added OA content of 0.01, 0.04, 0.10 and 0.4 mL.

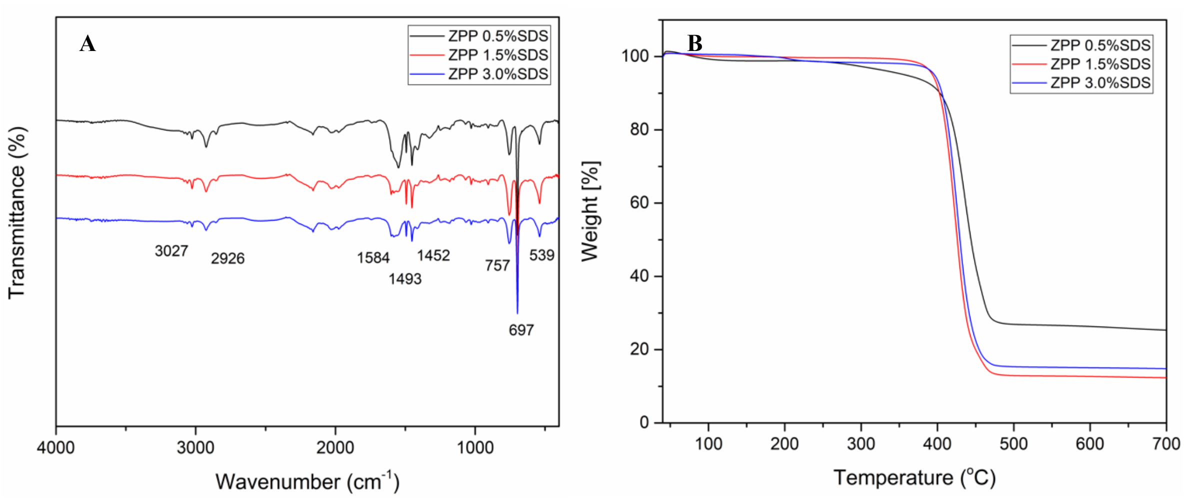

Figure S3. (A) FTIR spectra and (B) TGA thermograms of ZPPs prepared by varying the amount of SDS, i.e., 0.5 (black), 1.5 (red) and 3.0% (blue).

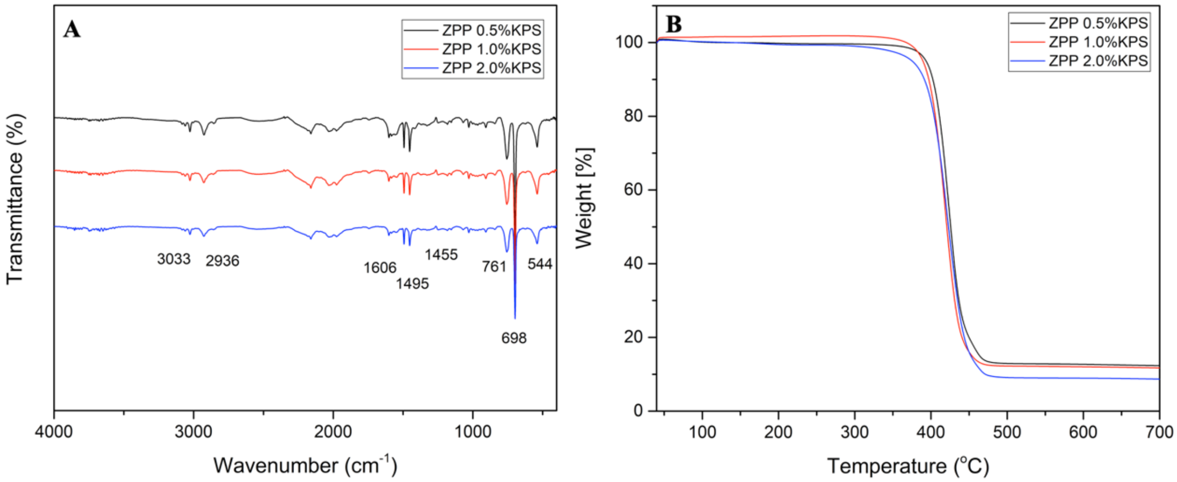

Figure S4. (A) FTIR spectra and (B) TGA thermograms of ZPPs prepared by varying the amount of KPS (0.5, 1.0 and 2.0%).

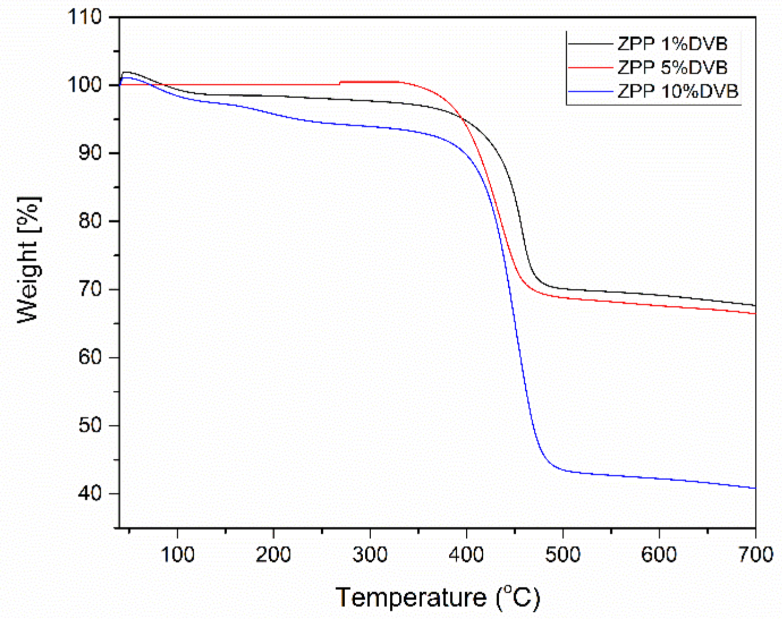

Figure S5. TGA thermograms of ZPPs using PMMA mixed with 1, 5 and 10% DVB.

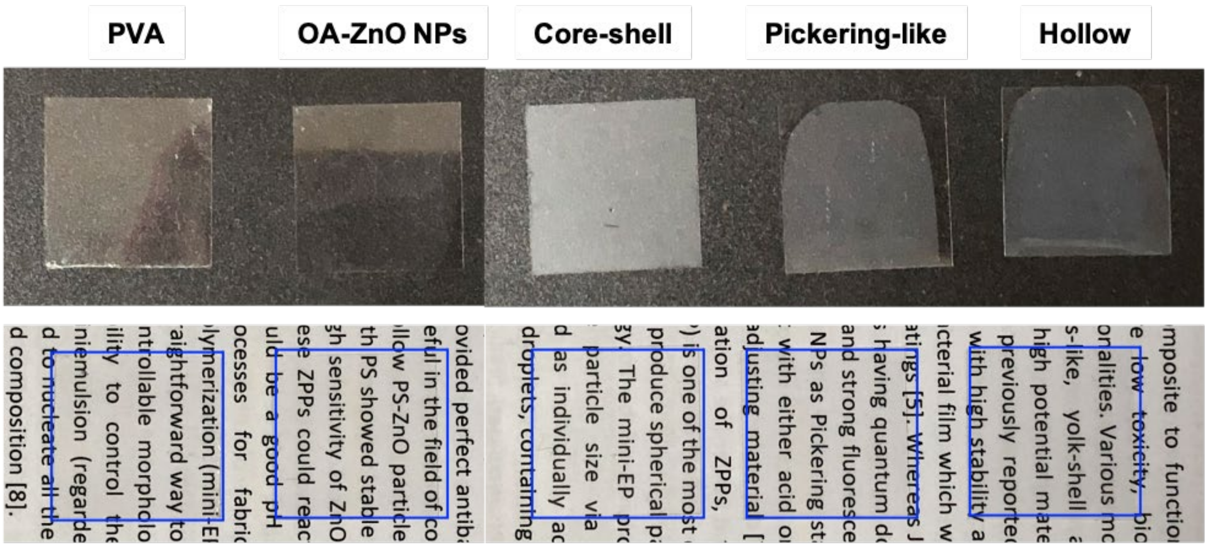

Figure S6. Photographs of PVA film and PVA films containing 1wt% of OA-ZnO NPs, core-shell ZPPs, Pickering-like ZPPs and hollow ZPPs coated on glass slide placed on black and white backgrounds.

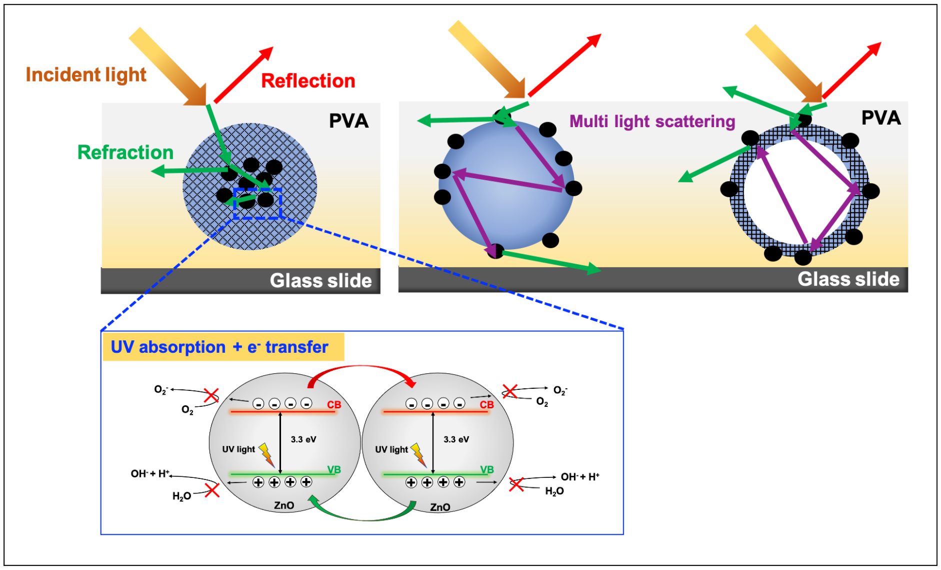

Figure S7. Schematic diagram of light absorption and multi light scattering of core-shell, Pickering-like and hollow ZPPs.
